# Progress of Surgery

**Published:** 1874-12

**Authors:** Jno. E. Owens

**Affiliations:** Lecturer on Surgery, Rush Medical College, Chicago


A MONTHLY RECORD OF

*Medicine, Surgery and the Collateral Sciences.*

EDITED BY J. ADAMS ALLEN, M.D., LL.D.; AND WALTER HAY, M.D.

VOL. XXXI. — DECEMBER, 1874. — NO. 12.

### Progress in Medical Sciences.

ARTICLE I.—*Progress of Surgery.* By JNO. E. OWENS, M.D., Lecturer on Surgery, Rush Medical College, Chicago.

1. (*a.*) Oakum for Drainage. (*b.*) Deformity after Fracture. Reported by Dr. PERRY, House Physician at Bellevue Hospital. (*The Medical Record*, New York, August 1, 1874.)

2. Treatment of Varices by Local Application of Perchloride of Iron. By Dr. LINON, of Verviers. (*The Lancet*, *The Medical Record*, New York, August 1, 1874.)

3. An Unnatural Position of the Head a Cause of Death from Anæsthetics. By Dr. G. W. COPELAND. (*The Medical Record*, New York, August 1, 1874.)

4. Result of Billroth's Case of Extirpation of the Larynx. (*The Boston Medical and Surgical Journal*, August 27, 1874.)

5. Surgical Treatment of Empyema. By HENRY CLARKE, M.D. (*The Boston Medical and Surgical Journal*, August 20, 1874.

1. (*a.*) For the purpose of securing free discharge from the bottom of deep wounds, after operation for excision of the hip-joint, for instance, the use of oakum is advocated. It is claimed that oakum will permit free discharge, and, at the same time, help to absorb it. It is regarded as of special importance in exsection of the hip-joint, to maintain firm compression about the wound, in order to give additional security against infiltration of the tissues with the discharge. This is best accomplished, after plugging the wound with the oakum, by covering the part with a thick layer of the same, and applying over all, with sufficient firmness, a roller bandage.

(*b.*) Deformity after fracture is very apt to occur, especially in the lower extremities, if the soft callus is not properly protected for some time after union has taken place. If supporting apparatus is not used when the patient commences to walk around, the soft material will commence to yield, and ugly deformity may result, much to the annoyance of both patient and surgeon.

2. Dr. Linon has used perchloride of iron, locally, with great success in the treatment of varices. The strength of the solution is two and a half drachms to eight ounces of water.

Compresses of flannel are steeped in the solution, then wrung out and applied by means of a flannel bandage, which is only moderately tightened. This application is to be kept on twenty-four hours, and, upon its removal, the surgeon will be much surprised to find that the venous dilatations have almost entirely disappeared. The applications are to be renewed, as above, during seven or eight days successively, after which time the bandage is to be kept on, without any further wetting, till it becomes loose. It is then to be wetted with the solution again, and applied until the varices have disappeared, which generally takes place after eight days or two weeks, according to the size of the swelling. Dr. Linon has been able to show that the success is to be attributed to the local action of the iron, and not to the compression.

3. Dr. Copeland's paper on the "Styloid Muscles and Anæsthetics," appeared in the Boston *Medical and Surgical Journal* of February, 1874. The cause of impeded breathing during anæsthesia was attributed to the action of the styloid muscles in closing the glottis. It was shown that the difficulty could always be relieved by simply tilting the head forward in order to relax these muscles, without making traction on the tongue. It is further claimed (Philadelphia *Medical Times*, May 30) that death from chloroform and other anæsthetics is often caused by an unnatural position of the head, the latter being thrown back and the styloid muscles put upon the stretch. The author endeavors to show that all deaths from nitrous oxide gas, and a large number of those from other anæsthetics, have taken place while the patients were in a sitting position, which position would allow the head to fall further back than if they were lying down. Further, deep inspirations through the mouth rapidly inflate the lungs, but they (the lungs) as rapidly become emptied, leaving a long interval during which there is but little vapor in the lungs. Inhalations through the nose are recommended, since it requires a much longer time to inflate the lungs, and a much longer time to empty them, leaving no interval.

4. Upon the authority of the *Medical Times and Gazette*, the journal referred to in the index records the death of Billroth's case of extirpation of the larynx. The operation was performed on the 31st of January last, and death occurred July 7th. The cancer, the disease which necessitated the operation, reappeared locally soon after the return of the patient to his home.

5. Dr. Clarke reports five cases of empyema, treated by means of free openings, viz.:

CASE I.—The puncture was made, with the small trocar of the Bowditch apparatus, in front, in the fifth intercostal space, midway between the axillary and mammary lines. Three pints of pus were pumped off. The patient improved for ten days. At the end of two weeks, however, he began to complain of increased oppression in breathing, to have more fever and perspiration, and the physical signs already indicated the re-accumulation of the fluid. The patient growing steadily worse, three weeks from the date of the first puncture, consent was obtained to make a free opening in the chest-wall. The point selected was the same selected for the former puncture. The opening gave exit to three pints of pus. A piece of soft linen was placed in the opening. The pleural cavity was washed, at first, with a solution of carbolic acid, one part of the acid to ninety-nine parts of water, and finally with one containing two teaspoonsful of liquid carbolic acid to a pint of water and one ounce of glycerine. By May 12th, about two months from the time of the operation, the discharge having nearly ceased and become more serous, the opening was allowed to close, which it did almost immediately. The patient continued to cough for nearly two months longer, but at last made a good recovery.

CASE 2.—The patient was an inmate of the City Hospital at Worcester, and had been ill five months. An inflamed tumor, fluctuating and tender, supervened at the left lower lateral chest. During an attack of vomiting he felt something give way in the locality of the lump, and soon after coughed up pus in sufficient quantity to fill both the mouth and nose. On the twenty-third day after his admission, a free incision was made in the tumor. Pus discharged freely, and the pleural cavity was washed out twice daily with carbolic acid. After three or four days the patient was able to taste the acid at each injection. A harassing cough was almost immediately relieved, the breathing became easy, and in three weeks from the date of the opening the discharge had become quite small in quantity. June 14th, the patient (admitted May 22d) was allowed to return home. Soon after, the wound quite healed, and he made a good recovery.

CASE 3.—This patient was a scrofulous child, aged four and a half years. Suppurative pleurisy followed scarlatina. This little girl recovered from a very extreme condition, and nothing but the surgical treatment employed could have saved her life. Soon after the case came under Dr. Clarke's care, he was summoned in haste to find the patient in a convulsion. Subsequently her pulse was 160, and the temperature fluctuated very much, ranging from 100° to 106°.

On the twenty-second day of the illness, more than a quart of pus was withdrawn by aspiration, the patient being under the influence of chloroform. Relief was immediate; the temperature fell, the cough lessened, and the appetite improved. Improvement, however, continued only four or five days, and at the end of a week after the tapping it was decided to make a free opening in the chest, in the fifth intercostal space, between the mammary and axillary lines. Fully a quart of pus escaped. The cavity was washed daily with the following solution: A teaspoonful of liquid carbolic acid and about an ounce of glycerine to a pint of blood-warm water.

Five weeks after the last operation, the discharge still being abundant, a solution of iodide of potassium, with tincture of iodine, was substituted for that of carbolic acid. The discharge then diminished more rapidly. Eight weeks from the time of the operation, the discharge growing quite thin, the wound was allowed to close. (We have not space for an outline of the other two cases. They all show the superiority of a free opening in cases of suppurative pleuritis over all other means of treatment. The improvement gained by tapping and by aspiration, is short-lived. Treatment by a free opening is curative.)

